# Who turns to the human? Companion pigs’ and dogs’ behaviour in the unsolvable task paradigm

**DOI:** 10.1007/s10071-020-01410-2

**Published:** 2020-07-17

**Authors:** Paula Pérez Fraga, Linda Gerencsér, Melinda Lovas, Dóra Újváry, Attila Andics

**Affiliations:** 1 Department of Ethology, Eötvös Loránd University (ELTE), Pázmány P. s. 1/C, Budapest, 1117 Hungary; 2 MTA-ELTE ‘Lendület’ Neuroethology of Communication Research Group, Hungarian Academy of Sciences, Eötvös Loránd University, Budapest, Hungary

**Keywords:** Comparative, Dog, Human–animal interaction, Interspecific communication, Pig, Unsolvable task

## Abstract

When facing an unsolvable problem, dogs exhibit spontaneous human-oriented behaviours (e.g. looking at the human partner, gaze alternations between the human and the target) sooner and for longer than domestic cats and hand-raised wolves. These behaviours have been interpreted as interspecific communicative acts aimed to initiate interaction. Here, we compare the emergence of human-oriented behaviours (e.g. orientation towards humans, orientation alternations, vocalizations) in similarly raised family dogs and miniature pigs utilising an unsolvable task paradigm which consists of Baseline (no task), Solvable and Unsolvable phases. Relative to the Baseline phase in which both species showed human-oriented behaviours to a similar extent, during the Unsolvable phase dogs showed more and pigs showed less such behaviours. Species-predispositions in communicative behaviour may explain why dogs have a higher inclination than pigs to initiate interspecific interactions with humans in problem-solving contexts.

**Electronic supplementary material:**

The online version of this article (10.1007/s10071-020-01410-2) contains supplementary material, which is available to authorized users.

## Introduction

Various domestic (Malavasi and Huber 2016; Miklósi et al. 2000; Nawroth et al. 2016; Turner 2017) and wild (Canteloup et al. 2015; Roberts et al. 2014; Xitco et al. 2004, 2001) mammals engage in communicative interactions with humans. Family dogs (*Canis familiaris*) may be unique in performing a variety of human-directed communicative behaviours (Kaminski and Nitzschner 2013; Udell and Wynne 2008; Udell et al. 2010) already from an early age (Passalacqua et al. 2011; Riedel et al. 2008). Dogs look at humans to establish joint attention (Bentosela et al. 2016; Miklósi et al. 2003) and they use gaze alternations for referential communication, analogously to human infants (Marshall-Pescini et al. 2013). Genetic changes during domestication may have predisposed dogs to communicate with humans more than other species (Hare et al. 2002; Sommese et al. 2019), although experience with humans during development also plays a role (Barrera et al. 2011; Marshall-Pescini et al. 2009).

When facing an unsolvable task, dogs looked at the human partner earlier and for longer periods than similarly socialized wolves (Marshall-Pescini et al. 2017a; Miklósi et al. 2003) and cats (Miklósi et al. 2005). Wolves and cats were more persistent in trying to solve the task independently (Marshall-Pescini et al. 2017a, b; Miklósi et al. 2005). Single-species studies also reported human-directed communicative behaviours in domestic farm animals (goats, horses) in similar contexts (Malavasi and Huber 2016; Nawroth et al. 2016). However, no direct comparisons have been made between the dog and another social domestic species kept in similar rearing conditions.

Like dogs, pigs (*Sus scrofa domesticus*) are also group-living, highly social animals (Marino and Christina 2015), performing a variety of intraspecific communicative signals (Bensoussan et al. 2019; Gieling et al. 2011). Similarly to dogs, pigs’ domestication, while clearly different in trajectory - dogs were used mainly for working purposes and pigs mainly as meat stock (Frantz et al. 2016), was also characterized by a relatively close human contact (Hongo 1998; Piper 2008) and occasionally, pigs were also used for work or treated as pets (Robbins and Rappaport 2006). Also today, miniature pigs are becoming popular as companion animals (Marino and Christina 2015), occupying a similar ‘social niche’ in human families as the family dog (Gerencsér et al. 2019). There has been growing interest in studying farm pigs’ interspecific social skills, focusing on their sensitivity to human communicative cues and attentive states (Albiach-Serrano et al. 2012; Bensoussan et al. 2019; Nawroth et al. 2013, 2014). Still, little is known about how pigs use communicative behaviours towards humans. Recently, we showed that even if kept as companion animals, pigs differ from dogs in their responses to human communicative cues (Gerencsér et al. 2019) and in exhibiting spontaneous human-oriented communicative behaviours. The two species differed in the readiness to look at the human face, a behaviour that dogs often performed in a neutral context but that was almost exclusively triggered in pigs in the presence of food. Pigs also vocalized more, and these results altogether indicated a strong influence of species-predispositions.

Here we compared human-directed communicative behaviours of ~ 7 months old dogs and miniature pigs—both kept as companion animals from an early age—in an unsolvable task paradigm. We hypothesized that both species would exhibit spontaneous human-oriented behaviours, and an increase of those behaviours during the unsolvable phase in comparison with a baseline phase, especially in dogs. We also expected more orientation alternations from dogs and more vocalizations from pigs, reflecting differential species-predispositions.

## Methods

### Subjects

Our subjects were juvenile pigs (*N* = 10, 6 males and 4 females, *X*_age_ ± SD = 7.0 ± 1.24 months, Minnesota and mixed miniature variants) and dogs (*N* = 12 family dogs passed the criteria out of 19 tested, see below), 7 males and 5 females, *X*_age_ ± SD = 6.91 ± 1.92 months, from 8 different breeds). All animals were living in human families from ~ 8 weeks of age (more details in Supplementary Table S1). Subjects from both species were tested intermixed, there was no fixed species order.

### Procedure

**Fig. 1:**
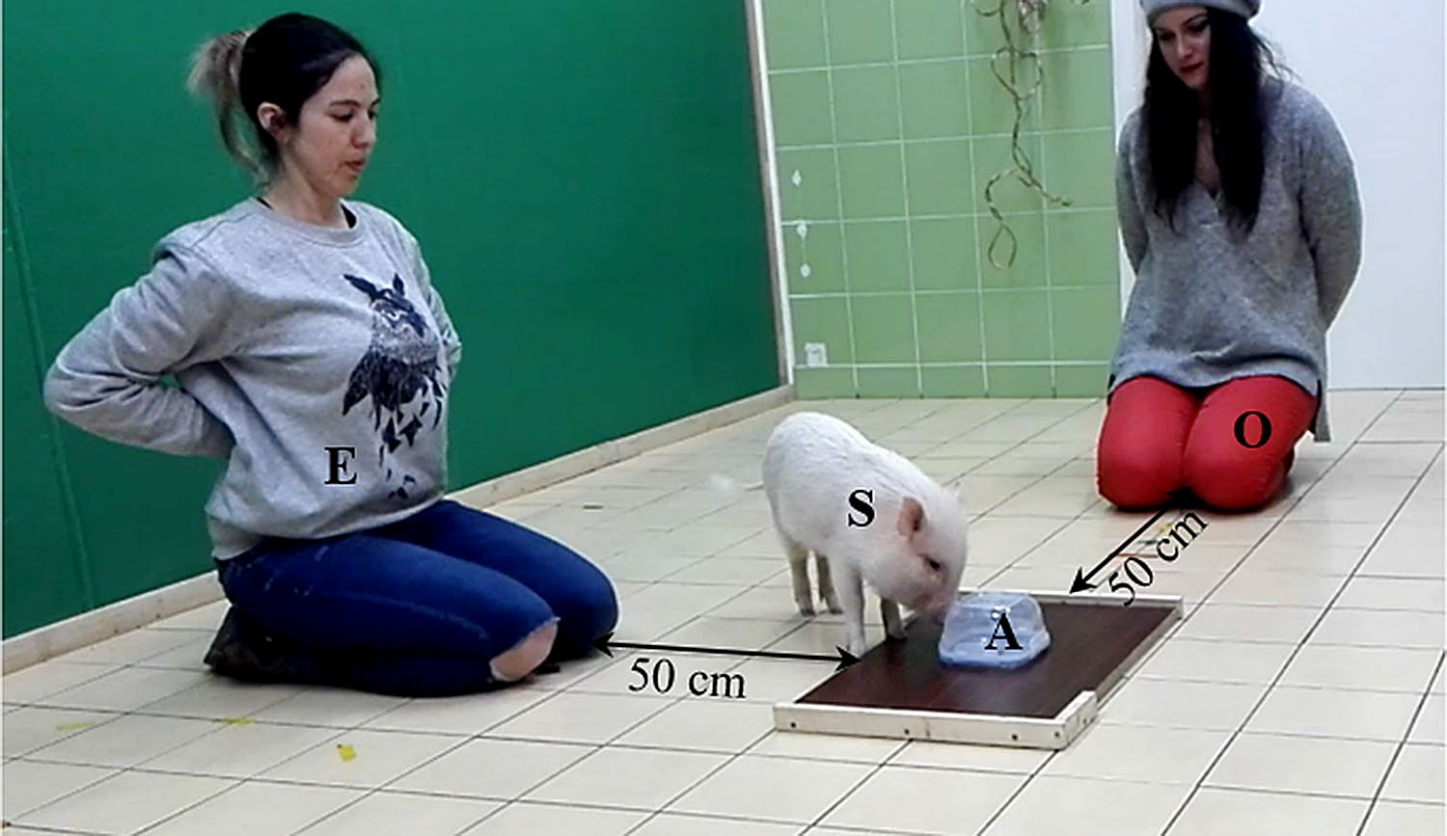
Experimental setup. *S* subject, *O* owner, *E* experimenter, *A* apparatus

The study was carried out in the laboratory (4.45 × 3.86 m room) of the Department of Ethology (Eötvös Loránd University, Budapest). A transparent plastic container (the apparatus, 15 × 15 cm) was placed equidistant from the two longer sides of the test room (Fig. 1), upside down (over a few titbits of sausages for dogs and apple/dog food for pigs—based on preparatory owner reports and pilot trials we assumed that these food types were of similarly high value for the individuals, since all of them willingly ate the offered titbits) on a wooden platform (40 × 60 cm), with the base permanently fixed to the platform. The upper cover part—with holes on it—could be moved off the platform easily by manipulation (solvable phase), but it could also be securely attached so the food was still visible but not accessible (unsolvable phase, adapted from Passalacqua et al. 2013).

Each individual test was done in the same day. We followed the method applied by Passalacqua et al. (2013) with some modifications; we increased the time of the unsolvable phase by 30 s to allow more time for the investigated behaviours to evolve, and we added an additional Baseline phase as well for observing any human- or apparatus-oriented behaviour in the absence of food, and familiarizing the subject (S) with the apparatus. The whole procedure consisted of a baseline phase (60 s), a solvable phase (5 solvable trials) and an unsolvable phase (90 s) in fixed order. Before the test session began, the S, the owner (O) and the experimenter (E) entered the test room and the S was allowed to walk around off leash and explore the room for 60 s.

During all phases O and E kneeled down by two different sides of the apparatus at 50 cm distance facing towards it. In the beginning of each phase O kept S in between his/her legs with S facing the apparatus. In the baseline phase, E manipulated the apparatus without covering it, placing the upper part next to the fixed bottom part. O let S free when E signalled with her hand. E and O, with the hands behind their backs stayed passive, following S with their gaze.

After a short (− 30 s) break, the test continued with the solvable phase. E showed a piece of food to S, placed it on the bottom of the apparatus without covering and O let S free. This served for testing S’s motivation for eating and informing the S where the food would be placed. E then showed another piece of food to S, placed it on the bottom of the apparatus and covered it with the upper part without closing it securely. O let S free. E and O did not move. The trial ended when S obtained the food or after a maximum 60 s. Only subjects succeeding (accessing the food) a minimum 3 out of 5 times (10/10 pigs, 12/19 dogs) were tested in the unsolvable phase that followed immediately, and included in analyses.

The unsolvable phase was identical to the solvable phase, except that the cover part of the apparatus was securely closed (i.e. food inaccessible for S).

### Behaviour coding

All tests were video recorded for behavioural analysis by Solomon Coder (v. 090913; © András Péter https://solomoncoder.com). Starting from O releasing S, during the baseline and unsolvable phases we measured latency and duration of orientation to humans, frequency of orientation-alternation between the apparatus and the humans, and durations of vocalization, human-oriented vocalization and apparatus-interaction. During the solvable phase we measured the latency to success (i.e. solving the task), and apparatus-interaction and human-orientation in the first trial (see Supplementary Table S2 for behavioural variables definitions).

The recordings were coded by one main coder, and twenty percent of them was also coded by a secondary coder. Interrater agreement was near perfect for ‘Success latency’ (ICC = 0.99 for both species). We used the raw coding sheets to calculate the agreement between the two raters for ‘orientation to human and to apparatus’ and ‘apparatus-interaction’, where the occurrence (yes/no) of any of these behaviours was marked every 0.2 s. The agreement was near perfect for ‘orientation’ (Cohen’s Kappa, *ĸ*pigs = 0.89, *ĸ*_dogs_ = 0.97, *Ps* < 0.001) and also for ‘apparatus-interaction’ (*ĸ*_pigs_ = 0.93, *ĸ*_dogs_ = 0.98, *Ps* < 0.001). We therefore used the coding of the main coder only to extract the variables of interest.

### Data analysis

We used the R statistical environment (v.3.5.0. R Development Core Team) with the following packages: lme4, emmeans and ggplot2. We used Shapiro–Wilk test and data visualization (normal Q-Q plots) to check for the distribution of the response variables and residuals, and applied Box-Cox power transformations with optimal lambda parameters where it was necessary to fulfil normality criteria. We used non-parametric tests where neither transformation method resulted in a normal distribution. We built a linear mixed-effects model (LMM) with ‘Success latency’ as the response variable, trial (within-subject) and species (between-subject) as explanatory factors and individual subjects as a random factor for the solvable phase analysis. We used Mann–Whitney–Wilcoxon tests to compare the total times pigs and dogs spent orienting at the two humans and manipulating the apparatus in the first trial of the solvable phase—to test for factors that could possibly explain success latency differences between the two species (see “Results”).

Since we did not aim to test for the familiarity effect of the humans on the subjects’ interspecific behaviour and the owner-experimenter contrast was not well controlled to make it clearly interpretable, we did not consider orientation to owner and experimenter as separate measures throughout the main analyses, but used a combined ‘orientation to human’ variable instead. To test for main effects of phase (baseline vs. unsolvable, within-subject factor) and species, as well as for their interaction, we built LMMs with either ‘orientation to human’ (duration, s), ‘latency of orientation to human’ (s), and ‘apparatus-interaction’ (duration, s) as response variables. For testing the same main and interaction effects on ‘orientation-alternation’ (Poisson-distributed count data) we built a generalized mixed-effects model (GLMM) fit by maximum likelihood using Laplace approximation. We included individual subjects as a random factor in all the models and obtained corrected multiple post-hoc comparisons for the fixed factors. In all the above models we used the data from the first 60 s of the unsolvable phase (and all data from the 60 s long baseline phase) to make a fair comparison between the two phases. However, to further explore species differences during the total duration of the unsolvable phase in the above mentioned response variables, we used Mann–Whitney–Wilcoxon tests and two-sample *t* test (according to data distribution). To show how the human-oriented behaviours in the unsolvable phase were divided across the experimenter and the owner, we built LMMs for ‘orientation’, ‘latency of orientation’, a GLMM for ‘orientation-alternation’, and tested for main effects of orientation target (experimenter and owner), species and their interaction (see Supplementary material).

We compared the number of pigs and dogs that vocalized in the baseline and unsolvable phase by Chi-square test (with Yates' continuity correction), and used Mann–Whitney–Wilcoxon test to compare the duration of their vocalization. Because of dogs’ overall less vocalization (see “Results”) we further analysed pigs’ vocal behaviour only. To see whether there was any difference between the proportions of time pigs vocalized while exhibiting different orientation behaviours, we calculated the ratios of ‘human- and apparatus-oriented vocalization’ and ‘orientation to human or apparatus’ in both conditions (to make a fair comparison between the proportions of times—expressed as the ratio of the total duration of the session—pigs spent vocalizing while being oriented either to the humans or to the apparatus), and compared them by Wilcoxon signed-rank tests.

## Results

In the solvable phase, animals’ performance improved significantly across the five trials (LMM, main effect of trial on ‘success latency’: *F*_4,84_ = 6.302, *P* < 0.001). Pigs proved to be overall faster than dogs (*X*_dogs_ ± SD = 12.8 ± 17.9 s and *X*_pigs_ ± SD = 6.5 ± 10.5 s; LMM, main effect of species on ‘success latency’: *F*_1,20_ = 5.188, *P* = 0.034). During the first solvable trial, a low proportion of the subjects showed any human-orientation (*N* = 2/10 pigs and *N* = 5/12 dogs)—measured from the moment they started manipulating the apparatus. The duration of human-orientation did not differ between the two species (*X*_Pigs_ ± SD = 1.0 ± 2.5 s, *X*_Dogs_ ± SD = 6.3 ± 12.2 s, *W* = 74, *P* = 0.282—although note the low subject number), while pigs spent significantly less time in total (*X*_Pigs_ ± SD = 4.1 ± 1.9 s) than dogs (*X*_Dogs_ ± SD = 9.9 ± 5.4 s) manipulating the apparatus (*W* = 103.5, *P* = 0.005) (note that *N* = 9/10 pigs and 9/12 dogs successfully opened the apparatus in the first trial, although all subjects attempted to).

**Fig. 2:**
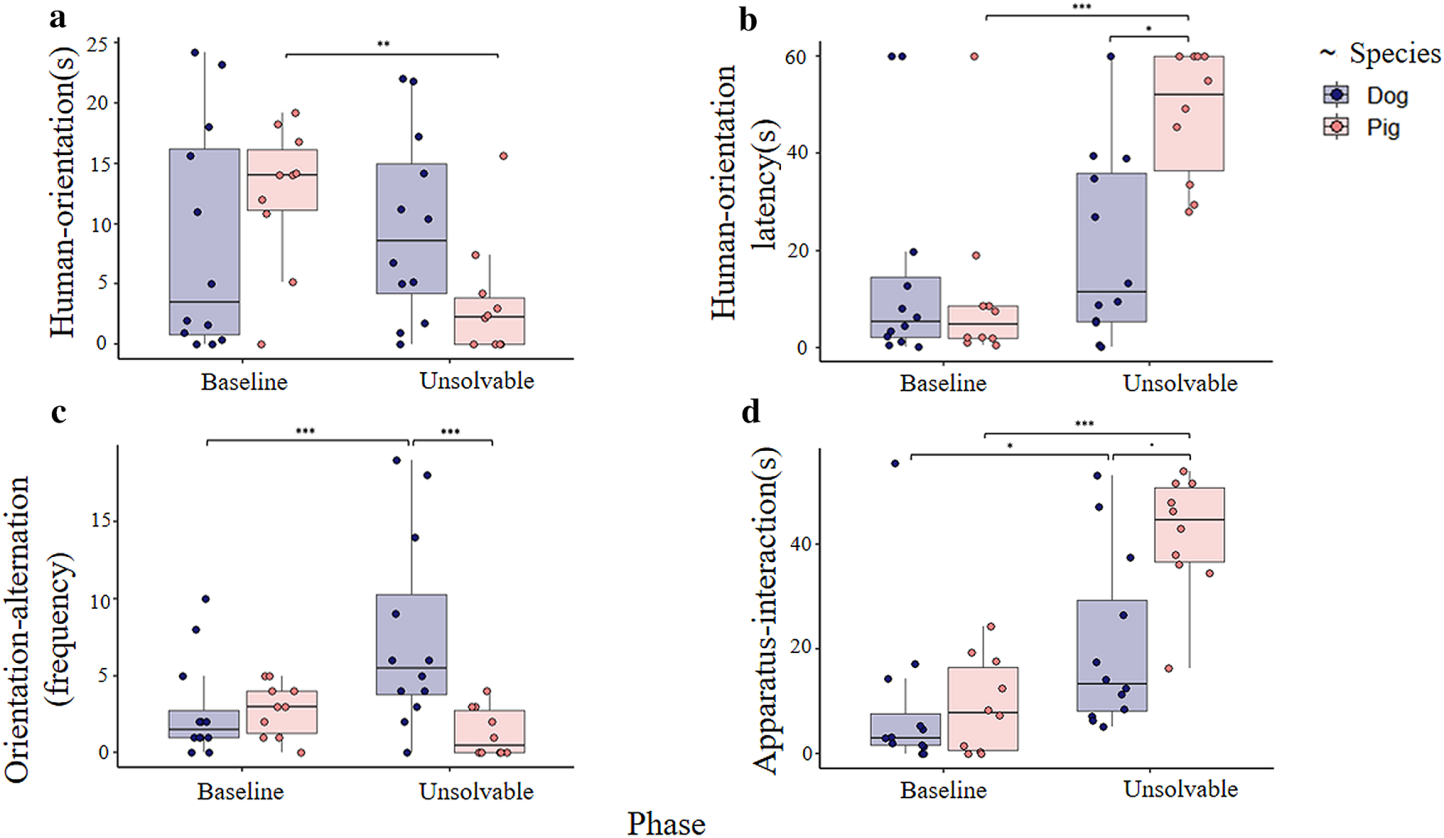
Pigs’ and dogs’ performance in the baseline and unsolvable (first 60 s) phases. Bold lines stand for the median, boxes indicate the interquartile range and whiskers extend until the smallest and largest values (excluding outliers and extremities). Dots represent individual data points. Significance codes of post-hoc comparisons: ***P < 0.001; **P < 0.01; *P < 0.05; ·*P* < 0.1 (see also Supplementary Tables S3–S10)

The joint analysis of the baseline and unsolvable phases revealed that the interaction between species and phase had a significant effect on several variables. Pigs in the unsolvable phase exhibited less human-orientation than in the baseline phase (interaction effect, LMM, *F*_1,20_ = 9.779, *P* = 0.005, Fig. 2a). Pigs oriented later to a social partner (either the owner or the experimenter) in the unsolvable than in the baseline phase, while dogs oriented sooner to a human than pigs in the unsolvable phase (interaction effect, LMM, *F*_1,20_ = 9.203, *P* = 0.007, Fig. 2b). Dogs in the unsolvable phase alternated their orientation more frequently between the apparatus and a human partner than in the baseline phase, and also more frequently than pigs (interaction effect, GLMM, *Z* = − 4.601, *P* < 0.001, Fig. 2c). Both species spent more time interacting with the apparatus in the unsolvable phase, and in this phase pigs interacted with the apparatus for longer than dogs (LMM, *F*_1,20_ = 4.426, *P* = 0.048, Fig. 2d). See Supplementary Tables S3–S10 for all corresponding post-hoc comparisons and further model parameters.

During the total duration of the unsolvable phase, dogs, as compared to pigs, spent more time orienting at humans (*X*_dogs_ ± SD = 14.4 ± 10.4 s, *X*_*p*igs_ ± SD = 5.8 ± 6.4 s; Mann–Whitney–Wilcoxon test, *W* = 92, *P* = 0.038), they also looked sooner (*X*_dogs_ ± SD = 21.9 ± 23.4 s, *X*_pigs_ ± SD = 57.8 ± 25.2 s; two-samples *t *test, *T*_18.68_ = − 3.429, *P* = 0.003) and exhibited more ‘orientation-alternations’ (*X*_dogs_ ± SD = 9.8 ± 7.4, *X*_pigs_ ± SD = 2.3 ± 2.3; Mann–Whitney–Wilcoxon test, *W* = 107, *P* = 0.002). Pigs spent more time than dogs interacting with the apparatus (*X*_pigs_ ± SD = 51.44 ± 17.9 s, *X*_dogs_ ± SD = 24.9 ± 20.9 s, *W* = 88, *P* < 0.001). The follow-up analysis comparing experimenter- and owner-oriented behaviours showed earlier and longer orientation towards the experimenter in both species and more frequent orientation alternations with the experimenter in pigs, but did not reveal effects that biased the above species differences (see Supplementary Fig. S1 and Tables S11–13).

3/12 dogs vs. 7/10 pigs vocalized in the baseline phase (*χ*^2^ = 2.825, *P* = 0.093), and 2/12 dogs vs. 7/10 pigs in the entire unsolvable phase (*χ*^2^ = 4.402, *P* = 0.036). Pigs also spent more time vocalizing in both phases (Mann–Whitney–Wilcoxon tests, *W*_Baseline_ = 31.5, *P* = 0.043 and *W*_Unsolv_ = 25.5, *P* = 0.012). Because dog vocalizations were rare, we further analysed pigs’ vocal behaviour only. Pigs vocalized more during the baseline than the unsolvable phase (*X*_Baseline_ ± SD = 9.2 ± 8.9 s, *X*_Unsolv_ ± SD = 1.2 ± 1.3 s; Wilcoxon signed-rank test, *W* = 40, *P* = 0.044), but there was no difference between the two conditions in the relative duration of neither human-oriented nor apparatus-oriented vocalizations (Wilcoxon signed-rank tests, *W*_Human_ = 6, *P* = 0.205 and *W*_Apparatus_ = 14, *P* = 0.529).

## Discussion

To the authors’ present knowledge this is the first study comparing human-directed communicative behaviour of two social domestic species kept as companion animals, in an unsolvable task paradigm; and the first research on pigs’ referential communicative abilities towards humans. In line with our hypothesis, the two species exhibited human-oriented behaviours to a similar extent during the initial baseline phase, indicating that the spontaneous display of these behaviours is not unique to dogs. Differences became apparent in the problem-solving context only. As we predicted, dogs performed more human-oriented behaviours than pigs; they oriented for longer and earlier to the humans, and also alternated their orientation between the human partners and the apparatus more frequently, corroborating previous research (Miklósi et al. 2005, 2003). Interestingly, the expected increase of referential communicative signals during the unsolvable phase in comparison with the baseline phase was only observed in dogs but not in pigs. Pigs, in contrast, interacted more with the apparatus than dogs. Dogs thus may be predisposed to use orientation alternations to communicate referentially towards humans for problem solving, even at an early age. This is supported by previous observations including dogs’ tendency to reduce their independent problem-solving behaviours in humans’ presence (Brubaker et al. 2017; Udell 2015), and dogs’ ability to use similar communicative signals towards both conspecifics and humans (Hare and Tomasello, 1999)—analogue to those used among, and thus easy to recognize by humans (review Marshall-Pescini et al. 2013; Udell and Wynne 2008).

One could argue that pigs may use not less but different, perhaps less easily recognizable communicative signals. Pigs indeed tended to use more vocalizations, and earlier we showed that pigs make more physical contact with humans in a similar food-requesting context than in a neutral condition (Gerencsér et al. 2019). Nevertheless, as neither the total duration of vocalizations or human-oriented behaviours (including touch), nor human-oriented or apparatus-oriented vocalizations increased in the unsolvable vs. the baseline phase, we cannot claim that that pigs used these for referential signalling (i.e. to direct the human’s attention to the apparatus). Furthermore, pigs not only performed less human-oriented behaviours in the unsolvable phase than dogs, but also interacted more with the apparatus, which might have contributed to their decreased readiness to interact with humans compared to dogs.

Another potential reason for species differences in human-oriented behaviours in the unsolvable phase may be that pigs’ laterally positioned eyes, wider viewing angle (Zonderland et al. 2008) and less flexible neck (Sack 1982) make them anatomically less predisposed to orient towards a human and to perform orientation alternations, and they also need to do it less than dogs for a comparable visual input. In support, short-headed dogs (with less lateral eyes) were found to gaze more at humans than long-headed dogs (Bognár et al. 2018). However, both wolves (with similar anatomy) and cats (with a flexible neck Graf et al. 1995; Zhang et al. 2014) were shown to perform less human-oriented behaviours in a problem-solving context than dogs (Brubaker et al. 2017; Miklósi et al. 2005). Furthermore, we showed earlier that pigs easily orient towards a human face in food-related task settings (Gerencsér et al. 2019). Horses and goats, with similarly lateral eyes (Broadwater et al. 2007; Hanggi and Ingersoll 2012), have also been reported to perform orientation alternations (Langbein et al. 2018; Malavasi and Huber 2016; Nawroth et al. 2016). So while pigs’ anatomical and sensory capacities may have influenced their human-oriented behaviours, it is improbable that these account for much of the species differences.

Pigs’ greater manipulative persistence may reflect their predisposition to solve problems independently (Gieling et al. 2011) as argued for other species (Brubaker et al. 2017; Marshall-Pescini et al. 2017a). However, we cannot exclude that the difference in persistence here is partly caused by specific task features. First, the box-opening problem may have been more natural to pigs who routinely use their snout for rooting (Studnitz et al. 2007; Tynes 2001). Consequently, pigs may have perceived the task as one that is solvable independently, without communicating with humans. Pigs were indeed faster than dogs in their box-opening speed during the Solvable trials with also spending less time in total than dogs manipulating the apparatus in the first trial already. Thus, even though we cannot exclude that human-oriented behaviours could have also influenced success latency here, total manipulation time alone sufficiently accounts for the species differences behind the time taken to open the apparatus. Future comparative studies should attempt to control for species differences in task naturalness (Kamil and Mauldin 1988). Second, species differences in persistence may have been influenced by pigs’ greater food motivation (Marino and Christina 2015). We cannot exclude this, but dogs also attempted to solve the problem in all cases, and also improved across trials, suggesting that motivation alone does not account for the behavioural difference.

A few factors may limit the interpretation of our results. Even though we supervised pigs’ daily home routines to ensure similar rearing conditions to the two species, pigs’ and dogs’ interactions and experiences with humans may have differed. Additionally, we had no control over the first 8 weeks’ socialization events and how they affected the appearance of human-directed communicative propensities—especially in pigs, where the lack of breed variability (see Supplementary Table S1) may as well restrict the generalizability of the present results. Furthermore, we cannot completely exclude that the reported changes in behaviour between the baseline and the unsolvable phase are not due to the necessarily fixed order of the conditions (to confront the subject with a novel apparatus and measure spontaneous human-oriented behaviours without any possible expectations of food reward). However, we believe that the fact that expressed species differences emerged in the unsolvable phase only makes it unlikely that those would be due to an order effect, but rather due to the introduction of a new salient stimulus (i.e. food reward, clearly attractive for both species). Finally, since it was not our aim to test for the effect of familiarity with the humans, the study was not well controlled for contrasting owner- versus experimenter-oriented behaviours. Collapsing across the two humans in the analysis could possibly mask existing species differences, but the fact that the main results were not biased by the split follow-up analysis makes this improbable. We speculate that no bias in the unsolvable phase towards owner-oriented behaviours (similarly to Aniello and Scandurra 2016; Scandurra et al. 2015), and bias towards experimenter-oriented behaviours in both species may be explained by the fact that the experimenter was the one manipulating the food and showing it to the subject during the solvable trials, thus the animals may have expected food/instructions from her rather than from the owner.

To sum up, we used the unsolvable task paradigm framework to shed more light on the factors that influence the human-directed communicative abilities of domestic animals. The found parallels between dogs’ and highly socialized miniature pigs’ human-oriented behaviour in a neutral context point to similar propensities for interspecific interaction, given a similar socialization background. However, the differences between the two species in the problem-solving context suggest an influence of species-predispositions in communicative behaviours on why dogs are more successful than other species in engaging in interspecific interactions with humans.

## Electronic supplementary material

Below is the link to the electronic supplementary material.

Supplementary file1 (DOCX 348 kb)

Supplementary file2 (XLSX 25 kb)

## Data Availability

All data generated and analysed during this study are included in this published article and its Supplementary Information file (see Supplementary Data).
