# Supplementary material for: Who turns to the human? Companion pigs’ and dogs’ behaviour in the unsolvable task paradigm
Source: Anim Cogn. 2020 Jul 17;24(1):33–40. doi: 10.1007/s10071-020-01410-2 (PMC7829225; doi:10.1007/s10071-020-01410-2)
Supplement: Supplementary file 1 — Supplementary file1 (DOCX 348 kb) [file 10071_2020_1410_MOESM1_ESM.docx]

**Supplementary Information for the manuscript entitled**

**‘Who turns to the human? Companion pigs’ and dogs’ behaviour in the unsolvable task paradigm’**

**Authors and affiliations**

Paula Pérez Fraga^1*^, Linda Gerencsér^1 2^, Melinda Lovas^1^, Dóra Újváry^1^, Attila Andics^1 2^

1 Department of Ethology, Eötvös Loránd University (ELTE), Hungary

2 MTA-ELTE ‘Lendület’ Neuroethology of Communication Research Group, Hungarian Academy of Sciences – Eötvös Loránd University, Budapest, Hungary

* Correspondence: pauliperezfraga@gmail.com

**Subject information**

The pigs were acquired from six different breeders in Hungary and are from different litters with at least one parent different. They are also part of a long-term scientific project (<http://etologia.elte.hu/en/lendulet-neuroethology-of-communication/>), which required strict a priori selection procedure of the owners who volunteered to cooperate with the Department of Ethology, Eötvös Loránd University. The adoption process itself was supervised and guidelines were provided for the piglets’ at-home handling, socialization, exposure and habituation to different environments, etc. in order to make the rearing environment as similar to that of a well-socialized family dog as possible. Most of the dogs’ owners were regular volunteers of the Family Dog Project (<https://familydogproject.elte.hu/>), and the socialization background of the dogs – as based on the owner’s report prior to enrolment – was similar to that of the pigs.

**Table S1. Subject information**

| **Subject ID** | **Species** | **Birth date** | **Test age (months)** | **Gender** | **Breed** |
| --- | --- | --- | --- | --- | --- |
| 1 | Pig | 23/10/2017 | 6 | female | Minnesota |
| 2 | Pig | 14/08/2017 | 7 | male | Minnesota Mixed |
| 3 | Pig | 23/10/2017 | 6 | male | Minnesota |
| 4 | Pig | 19/08/2017 | 6 | male | Minnesota |
| 5 | Pig | 15/05/2017 | 10 | female | Minnesota Mixed |
| 6 | Pig | 05/04/2018 | 7 | male | Minnesota Mixed |
| 7 | Pig | 23/08/2017 | 7 | male | Minnesota |
| 8 | Pig | 25/04/2017 | 7 | female | Minnesota |
| 9 | Pig | 29/09/2017 | 6 | male | Minnesota |
| 10 | Pig | 25/03/2017 | 8 | male | Minnesota |
| 11 | Dog | 20/06/2018 | 7 | male | Alaskan Malamute |
| 12 | Dog | 05/01/2018 | 6 | female | Mixed |
| 13 | Dog | 06/01/2018 | 9 | male | Dachshund |
| 14 | Dog | 01/01/2018 | 6 | female | Mixed |
| 15 | Dog | 04/10/2017 | 6 | male | Beauceron |
| 16 | Dog | 27/04/2018 | 6 | male | Whippet |
| 17 | Dog | 27/06/2018 | 6 | male | Pumi |
| 18 | Dog | 05/11/2017 | 5 | female | Beagle |
| 19 | Dog | 15/05/2017 | 12 | male | Labradoodle |
| 20 | Dog | 02/05/2018 | 6 | female | Doberman |
| 21 | Dog | 20/03/2018 | 6 | male | Moscow Watchdog |
| 22 | Dog | 28/03/2018 | 8 | female | Border Collie |

**Behavioural variables**

**Table S2. Behavioural variables measured during the Baseline, Solvable and Unsolvable phases**

| **Behavioural Variables** | **Definition** |
| --- | --- |
| Orientation^*^ to human (duration, s)  (*Baseline* and *Unsolvable phase*) | S orients its head towards E or O from a static position or meanwhile moving, with or without establishing physical contact with the human |
| Latency of orientation to human (s)  (*Baseline* and *Unsolvable phase*) | The time taken (s) from the moment that S is released by O till it first orients its head to E or O |
| Orientation-alternation (frequency)  (*Baseline* and *Unsolvable phase*) | Orienting at the apparatus (i.e. S orients its head towards the apparatus from a static position or meanwhile moving) followed or preceded within a maximum of 3 s by orientating towards a human (O or E) |
| Apparatus-interaction (duration, s)  (*Baseline* and *Unsolvable phase*) | S establishes physical contact with the apparatus (nosing, licking, pawing, etc.) – referred to as persistence in the Unsolvable condition |
| Vocalization (duration, s) (*Baselin*e and *Unsolvable phase*) | S is vocalizing |
| Human-oriented vocalization (frequency)  (*Baseline* and *Unsolvable phase*) | Concurrence of Vocalization and Orientation to E or O (with or without interaction) |
| Apparatus-oriented vocalization (frequency)  (*Baseline* and *Unsolvable phase*) | Concurrence of Vocalization and Orientation to the apparatus (with or without interaction) |
| Success (latency, s)  (*Solvable* *phase*) | The time taken from the moment that S is released by O till it is opening the upper part of the apparatus for the last time before gaining access to the food |

^*^Note: We decided to report orientation, but not gazing direction (a variable commonly measured in the Unsolvable tasks studies, e.g. Passalacqua et al. 2011) or physical interaction (a variable measured in an earlier study comparing dog and pig behaviours, Gerencsér et al. 2019), for the following reasons. Gazing direction: Pigs’ anatomy (i.e. laterally positioned, relatively small eyes) made it difficult to accurately assess their gazing direction, whereas orientation allowed for a fairer species comparison. Physical interaction: During the Baseline phase 7/12 dogs and 9/10 pigs interacted physically with any of the humans, whereas only 3/12 and 2/10 pigs did so in the Unsolvable phase. Due to the lack of data for many subjects in the Unsolvable phase, this variable did not prove to be suitable for neither between-phase, nor between-species comparisons in the Unsolvable phase. On the other hand, the orientation variable (allowing for any physical interaction as well) gave us a good opportunity to carry out both within- and between-species comparisons.

**Post-hoc comparisons and model parameters**

**Table S3. *P* values and related parameters for the main effects and their interaction on ‘Orientation to human’ (LMM)**

| **Fixed effects** | **Estimate** | **Std. Error** | ***t* value** | ***P* value** |
| --- | --- | --- | --- | --- |
| Species  Phase  Species * Phase | 3.940  1.217  -10.177 | 3.152  2.194  3.254 | 1.250  0.555  -3.127 | 0.220  0.585  0.005 ^**^ |

Note. Significance code: '**' *P* < 0.01.

**Table S4. *P* values and related parameters of the post-hoc tests for the contrasts on ‘Orientation to human’** (Base: *Baseline phase*; Unsolv: *Unsolvable phase*)

| **Contrast** | **Estimate** | **SE** | **df** | **t ratio** | ***P* value** |
| --- | --- | --- | --- | --- | --- |
| Dog, Base- Pig, Base  Dog, Base- Dog, Unsolv  Pig, Base - Pig, Unsolv  Dog, Unsolv - Pig, Unsolv | -3.940  -1.217  8.960  6.237 | 3.152  2.194  2.403 | 32.84  20  20 | -1.250  -0.555  3.728 | 0.600  0.944  0.007 ^**^ |
|  |  | 3.152 | 32.84 | 1.978 | 0.217 |

Note. Significance code: '**' *P* < 0.01; Tukey method for *P* value adjustment.

**Table S5. P values and related parameters for the main effects and their interaction on ‘First orientation to human’ (LMM)**

| **Fixed effects** | **Estimate** | **Std. Error** | ***t* value** | ***P* value** |
| --- | --- | --- | --- | --- |
| Species  Phase  Species * Phase | -0.052  0.176  0.572 | 0.177  0.127  0.189 | -0.294  1.387  3.034 | 0.77  0.181  0.007 ^**^ |

Note. Significance code: '**' *P* < 0.01.

**Table S6. *P* values and related parameters of the post-hoc tests for the contrasts on ‘First orientation to human’** (Base: *Baseline phase*; Unsolv: *Unsolvable phase*)

| **Contrast** | **Estimate** | **SE** | **df** | **t ratio** | ***P* value** |
| --- | --- | --- | --- | --- | --- |
| Dog, Base - Pig, Base  Dog, Base - Dog, Unsolv  Pig, Base- Pig, Unsolv  Dog, Unsolv - Pig, Unsolv | 0.052  -0.176  -0.748  -0.520 | 0.176  0.127  0.139 | 33.76  20  20 | 0.294  -1.387  -5.374 | 0.991  0.521  0.0002 ^***^ |
|  |  | 0.176 | 33.76 | -2.954 | 0.028 ^*^ |

Note. Significance codes: '***' *P* < 0.001; '*' *P* < 0.05. Tukey method for *P* value adjustment.

**Table S7. *P* values and related parameters for the main effects and their interaction on ‘Orientation alternation’ frequency (GLMM)**

| **Fixed effects** | **Estimate** | **Std. Error** | ***z* value** | ***P* value** |
| --- | --- | --- | --- | --- |
| Species  Phase  Species * Phase | 0.167  1.003  -1.771 | 0.376  0.201  0.385 | 0.444  4.998  -4.601 | 0.657  < 0.0001 ^***^  < 0.0001 ^***^ |

Note. Significance code: '***' *P* < 0.001.

**Table S8. *P* values and related parameters of the post-hoc tests for the contrasts on ‘Orientation alternation’ frequency** (Base: *Baseline phase*; Unsolv: *Unsolvable phase*)

| **Contrast** | **Estimate** | **SE** | **df** | ***z* ratio** | ***P* value** |
| --- | --- | --- | --- | --- | --- |
| Dog, Base - Pig, Base  Dog, Base - Dog, Unsolv  Pig, Base - Pig, Unsolv  Dog, Unsolv - Pig, Unsolv | -0.167  -1.003  0.767  1.604 | 0.376  0.201  0.328 | Inf  Inf  Inf | -0.444  -4.998  2.337 | 0.971  < 0.0001 ^***^  0.09 |
|  |  | 0.402 | Inf | 3.985 | 0.0004 ^***^ |

Note. Significance code: '***' *P* < 0.001. Tukey method for *P* value adjustment.

**Table S9. *P* values and related parameters for the main effects and their interaction on ‘Apparatus interaction’ (LMM)**

| **Fixed effects** | **Estimate** | **Std. Error** | ***t* value** | ***P* value** |
| --- | --- | --- | --- | --- |
| Species  Phase  Species * Phase | 0.115  1.323  1.167 | 0.486  0.374  0.555 | 0.236  3.536  2.104 | 0.815  0.002 ^**^  0.048 ^*^ |

Note. Significance codes: '**' *P* < 0.01; '*' *P* < 0.05.

**Table S10. *P* values and related parameters of the post-hoc tests for the contrasts on ‘Apparatus-interaction’** (Base: *Baseline phase*; Unsolv: *Unsolvable phase*)

| **Contrast** | **Estimate** | **SE** | **df** | **t ratio** | ***P* value** |
| --- | --- | --- | --- | --- | --- |
| Dog, Base - Pig, Base  Dog, Base - Dog, Unsolv  Pig, Base - Pig, Unsolv  Dog, Unsolv - Pig, Unsolv | -0.115  -1.323  -2.409  -1.282 | 0.486  0.374  0.41 | 35.68  20  20 | -0.236  -3.536  -6.077 | 0.995  0.01 ^*^  < 0.0001^***^ |
|  |  | 0.486 | 35.68 | -2.639 | 0.057 . |

Note. Significance codes: '***' *P* < 0.001; '*' *P* < 0.05; ‘.’ *P* < 0.1. Tukey method for *P* value adjustment.

**Table S11. *P* values and related parameters of the post-hoc tests for the contrasts on ‘Orientation’ *(Unsolvable phase)*** (Exp: Experimenter)

| **Contrast** | **Estimate** | **SE** | **df** | **t ratio** | ***P* value** |
| --- | --- | --- | --- | --- | --- |
| Dog, Exp - Pig, Exp  Dog, Exp - Dog, Owner  Pig, Exp - Pig, Owner  Dog, Owner - Pig, Owner | 0.159  0.052  0.204  0.311 | 0.102  0.052  0.204 | 34.75  20  20 | 1.556  0.683  2.445 | 0.416  0.902  0.101 |
|  |  | 0.311 | 34.75 | 3.043 | 0.022 ^*^ |

Note. Significance codes: '***' *P* < 0.001; '*' *P* < 0.05; ‘.’ *P* < 0.1. Tukey method for *P* value adjustment.

**Table S12. *P* values and related parameters of the post-hoc tests for the contrasts on ‘Orientation latency’ *(Unsolvable phas*e)** (Exp: Experimenter)

| **Contrast** | **Estimate** | **SE** | **df** | **t ratio** | ***P* value** |
| --- | --- | --- | --- | --- | --- |
| Dog, Exp - Pig, Exp  Dog, Exp - Dog, Owner  Pig, Exp - Pig, Owner  Dog, Owner - Pig, Owner | -36.757  -15.083  -14.200  -35.873 | 10.742  7.776  8.519 | 33.92  20  20 | -3.422  -1.940  -1.667 | 0.009 ^**^  0.244  0.366 |
|  |  | 10.742 | 33.92 | -3.340 | 0.011 ^*^ |

Note. Significance codes: '**' *P* < 0.01; '*' *P* < 0.05. Tukey method for *P* value adjustment.

**Table S13. *P* values and related parameters of the post-hoc tests for the contrasts on ‘Orientation-alternation’ *(Unsolvable phase)*** (Exp: Experimenter)

| **Contrast** | **Estimate** | **SE** | **df** | **z ratio** | ***P* value** |
| --- | --- | --- | --- | --- | --- |
| Dog, Exp - Pig, Exp  Dog, Exp - Dog, Owner  Pig, Exp - Pig, Owner  Dog, Owner - Pig, Owner | 1.130  0.377  1.558  2.311 | 0.3920.187  0.550 | Inf  Inf  Inf | 2.886  2.013  2.832 | 0.020 ^*^  0.183  0.024 ^*^ |
|  |  | 0.598 | Inf | 3.866 | 0.0006 ^***^ |

Note. Significance codes: '***' *P* < 0.001; '*' *P* < 0.05. Tukey method for *P* value adjustment.

**
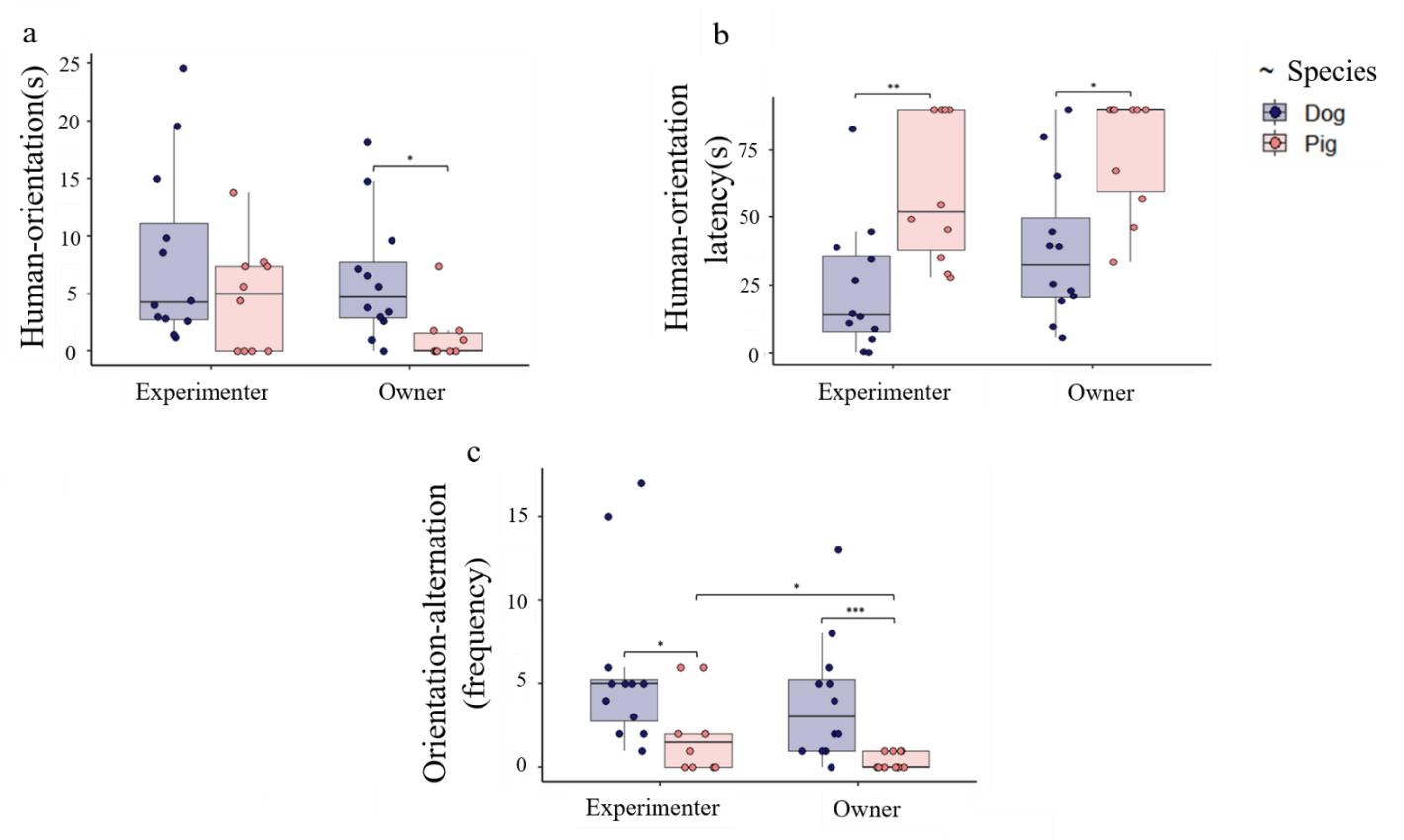
Fig. S1** **Pigs’ and dogs’ experimenter vs. owner oriented behaviours during the Unsolvable phase (90 s)**. Bold lines stand for the median, boxes indicate the interquartile range and whiskers extend until the smallest and largest values (excluding outliers and extremities). Dots represent individual data points. Dogs spent more time than pigs orienting at the owner and the experimenter (main effect of Species, LMM, F_1,20_=7.617, P=0.012), and the two species oriented more to the experimenter (main effect of Orientation target, LMM, F_1,20_=5.138, P=0.035). Dogs oriented sooner to both humans (main effect of Species, LMM, F_1,20_=16.058, P<0.001), while the two species oriented sooner to the experimenter than to the owner (main effect of Orientation target, LMM, F_1,20_=6.446, P=0.019). Dogs exhibited more experimenter-apparatus and owner-apparatus orientation-alternations than pigs, and pigs alternated their orientation more frequently between the experimenter and the apparatus than the owner and the apparatus (interaction effect, GLMM, Z=-2.032, P=0.042). Significance codes of post-hoc comparisons: '***' P<0.001; ‘**' P<0.01; '*' P<0.05 (see also Tables S11-S13)

**References**

Gerencsér, L, Pérez Fraga, P, Lovas, M., Újváry, D, Andics, A (2019) Comparing interspecific socio-communicative skills of socialized juvenile dogs and miniature pigs. Anim. Cogn. 1–13. https://doi.org/10.1007/s10071-019-01284-z

Passalacqua, C, Marshall-Pescini, S, Barnard, S, Lakatos, G, Valsecchi, P, Prato, E (2011) Human-directed gazing behaviour in puppies and adult dogs , Canis lupus familiaris. Anim. Behav. 82, 1043–1050. https://doi.org/10.1016/j.anbehav.2011.07.039
